# Intraspinal vascular perfusion territories of the descending thoracic aorta

**DOI:** 10.1093/ejcts/ezaf212

**Published:** 2025-06-27

**Authors:** Paata Pruidze, Giorgi Didava, Jeremias T Weninger, Stefan H Geyer, Christoph Neumayer, Josif Nanobachvili, Wolf Eilenberg, Martin Czerny, Wolfgang J Weninger

**Affiliations:** Division of Anatomy, Medical University of Vienna, Vienna, Austria; Division of Anatomy, Medical University of Vienna, Vienna, Austria; Division of Anatomy, Medical University of Vienna, Vienna, Austria; Division of Anatomy, Medical University of Vienna, Vienna, Austria; Division of Vascular Surgery, Medical University of Vienna, Vienna, Austria; Division of Vascular Surgery, Medical University of Vienna, Vienna, Austria; Division of Vascular Surgery, Medical University of Vienna, Vienna, Austria; Department of Cardiovascular Surgery, University Heart Center Freiburg - Bad Krozingen, Freiburg, Germany; Faculty of Medicine, Albert Ludwig University Freiburg, Freiburg, Germany; Division of Anatomy, Medical University of Vienna, Vienna, Austria

**Keywords:** Aneurysm, Surgery, Stent graft, Wall dissection, Spinal cord

## Abstract

**OBJECTIVES:**

Our study aims at characterizing the intraspinal vascular perfusion territories (angiosomes) of the descending thoracic aorta in a cadaver stetting to understand the principles of blood supply to the spinal cord and to provide the anatomic basis for strategies to avoid spinal ischaemia during aorta surgery.

**METHODS:**

Simulating blood flow in the descending thoracic aorta and thoracic aortic segmental arteries T3–T11 of 8 body donors were perfused with dyed liquids to label the epidural and spinal cord angiosomes.

**RESULTS:**

The cranial and caudal borders of the spinal cord angiosome varied substantially with a maximum extension from segment C7 to the conus medullaris. In 5 specimens, the anterior and posterior aspects differed for 1–2 segments. In 75%, the anterior spinal artery appeared to be stained along the entire spinal cord, and in 4 specimens, a voluminous Adamkiewicz artery joined its lower thoracic segments. In 3 of those specimens, this caused the spinal cord angiosome to be stained caudally towards the conus medullaris. In addition to details on the spinal cord angiosome, details on the epidural angiosome and the antero- and retrograde perfusion of the spinal nerve roots and the influence of thoracic aortic segmental artery variations are provided.

**CONCLUSIONS:**

Our study characterizes both intraspinal descending thoracic aorta angiosomes. It demonstrates the importance of the Adamkiewicz and the anterior spinal arteries for blood supply to the spinal cord and the nerve root fibers.

## Graphical Abstract

**Figure:**
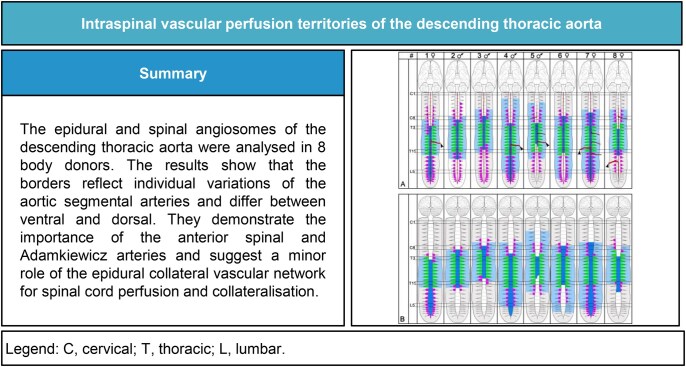

## INTRODUCTION

The human aorta is an organ and has 2 major sections [1]: The thoracic aorta in the thorax and the abdominal aorta in the retroperitoneum. From proximal to distal, the segments of the thoracic aorta are the ascending aorta, aortic arch and descending aorta. Beginning with the level of the third thoracic vertebra (T3), the thoracic descending and continuing abdominal aorta form thoracic- and lumbar aortic segmental arteries (TASAs; LASAs), which supply the tissues of the respective body segments including the spinal cord.

Recently it became evident that TASAs and LASAs show a high variability. Therefore, precise knowledge on aortic segmental artery patterns is essential to understand the individual situation regarding the risk of spinal cord injury during aorta surgery [2]. Of similar importance is information on the spinal cord volume, that aortic segmental arteries and aorta segments effectively supply. However, up to now no study examines the spinal cord volume supplied by selected single aortic segmental artery or of the sum of aortic segmental arteries arising from entire aorta segments. Information on the latter would also permit researching the efficiency of the networks of collateral channels in the epidural and subarachnoid spaces [3]. Such information can serve as a benchmark for researching the effect of blockade of aortic segmental artery origins and for planning save surgical interventions in the descending aorta.

Tissue volumes, to which blood is supplied to via a defined blood vessel or the sum of branches of a defined blood vessel segment, are named as ‘angiosomes’ [4]. Since blood vessels feeding an angiosome might reach various anatomic structures, various types of angiosomes can be distinguished. As an example, inside the spinal canal, an angiosome of the epidural space and the dura, and an angiosome of the subarachnoid space and spinal cord are evident.

This study aims at determining the maximal cranio-caudal extensions of the various intraspinal angiosomes of the descending segment of the thoracic aorta down to T11 in an elderly population by simulating blood flow under largely regular conditions in a human cadaver setting.

## MATERIALS AND METHODS

### Study design

The extension of the intraspinal vascular perfusion territories of 8 human body donor cadavers (4 males, 4 females) with an average age of 84 years (74–95 years) and a mean body mass index (BMI) of 19.7 (15.1–26.3) was examined by perfusion of coloured liquid in a post-mortem setting.

### Ethics

The study was conducted at the Division of Anatomy, Medical University of Vienna. All body donors included in this study had provided written consent to donate their dead bodies for teaching and science. In addition, all study protocols had been approved by the local ethics committee (EC Nr: 1748/2021).

### Anatomical investigation

Eight body donors delivered between 6 and 15 hours after death were investigated at room temperature (20–30°C). Using traditional anatomic approaches [5, 6], the thorax and abdomen were opened, the organs and diaphragm were removed, and the dorsal mediastinum and retroperitoneal spaces were approached, by removing the organs and tissues. The branches arising from the anterior and lateral sides of the aorta were tightly ligated and cut and the aorta, iliac and subclavian arteries were fully exposed. The aortic segmental arteries of the descending thoracic aorta (DTA) were followed for a distance of 5 cm to identify individual variations.

**Figure 1:**
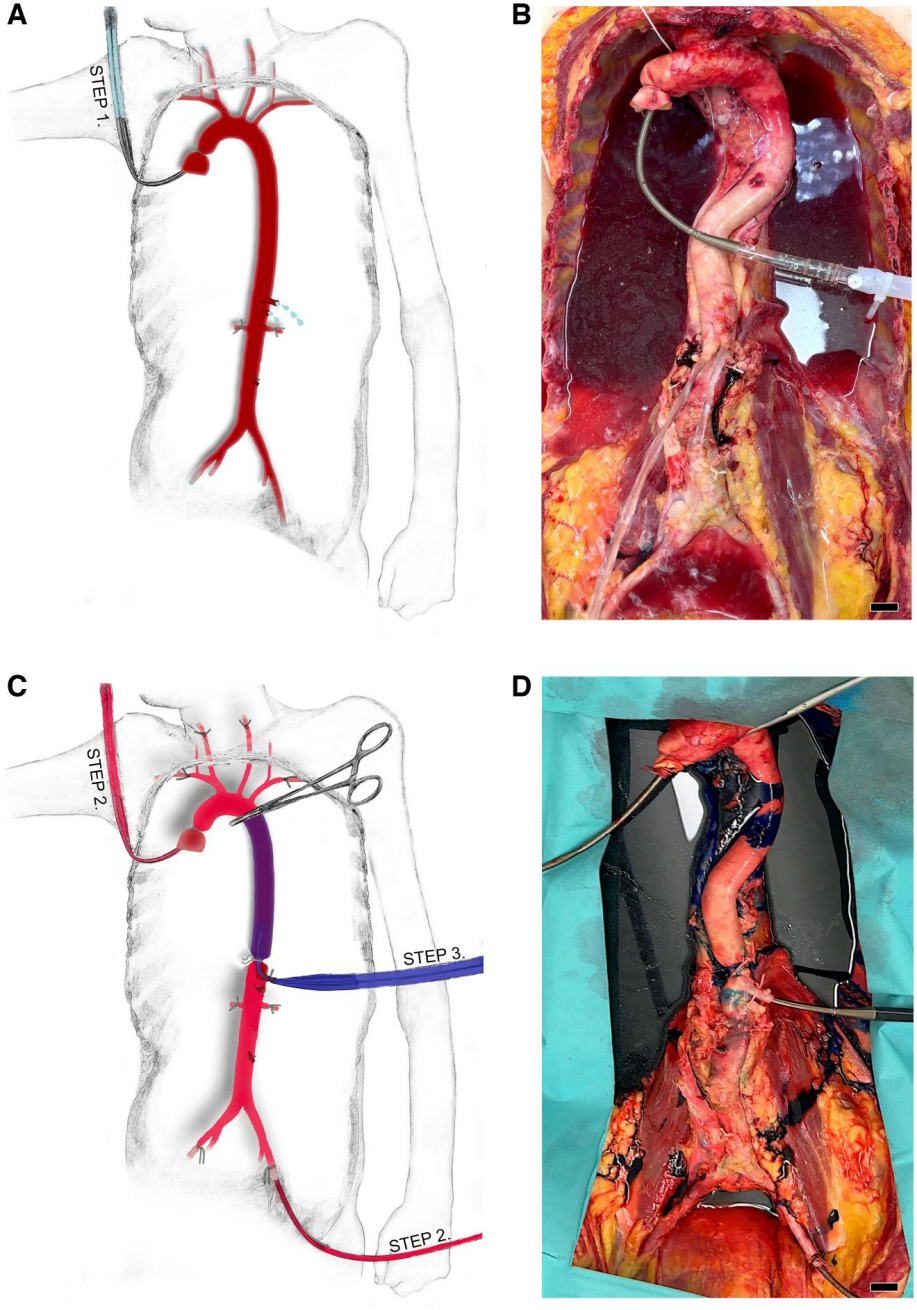
Perfusion setting. (**A**, **B**) Setting for flushing the aorta and arteries with NaCl 0.9%. Note the cannula in the ascending aorta. (**C**, **D**) Setting for perfusion with dyed liquid. Note the additional cannula in the left external iliac artery and the coeliac trunk. Scale bars, 2 cm.

Then the ascending aorta was cut. A 17 Fr Bio-Medicus™ femoral cannula (Medtronic, Minneapolis, MN, USA) was inserted, and the vessel wall was firmly attached to it. Via this tube, a saline solution (NaCl 0.9%; 2500 ml) was infused in order to flush the blood out of the vessels (Fig. 1). The backflow of liquid from both venae cavae and the azygos vein was considered as a positive sign for permeability of at least the major blood vessels (Fig. 1).

For investigating the DTA angiosomes, a specialized occlusion-perfusion model was established. The aorta was cross-clamped immediately distal to the origin of the left subclavian artery, and a 15 Fr Bio-Medicus cannula (Medtronic, Minneapolis, MN, USA) was inserted in the coeliac trunk. It was pushed proximally into the thoracic aorta and secured with a ligature midway between the origin of the 11th intercostal and subcostal arteries. Another 17 Fr Bio-Medicus (Medtronic, Minneapolis, MN, USA) cannula was inserted in the left external iliac artery, and the vessel wall was tightly attached. Finally, the right external iliac artery was clamped (Fig. 1).

The shutters of the proximal-most and distal-most cannulas were opened simultaneously, and in 6 specimens, 2500–3000 ml eosin (0.1%) solution and in 2 specimens, methylene blue (1%) solution were allowed to drain into the ascending aorta and the external iliac artery. After 2 min, the shutters were secured, and in 6 specimens, 2500–3000 ml methylene blue (1%) and in 2 specimens, eosin (0.1%) solution were allowed to drain into the DTA through the coeliac artery. Both solutions roughly mimicked the rheological properties of blood [7]. All perfusions were conducted with passive statical pressure of 90 mmHg for a time period of 2 min (Fig. 1).

After perfusion, the cadaver was placed in prone position. The skin of the back and the fascia and muscles superficial to the vertebral column were removed. The spinal canal was opened, the spinal nerves were cut at the level of the intervertebral foramina, and the spinal cord and meninges were cut at the level of the occipital foramen. The entire spinal cord with ensheathing meninges, spinal nerves and spinal ganglia was extracted and placed, ventral downwards on a table. The dura was opened in the midline and folded laterally. In a coronary plane, the spinal cord was longitudinally split. The staining patterns of the dura mater and inside and outside of the spinal cord were examined and carefully documented. Using the spinal nerve root fibers as landmarks, the precise cranial and caudal borders of the spinal cord angiosome were defined independently by 2 scientists.

### Definitions

- ‘Reasonable time frame for perfusion’: building on our expertise in body dissection and perfusion, we defined post-mortem intervals of less than 15 hours as reasonable time frame for achieving high-quality perfusion results;
- ‘Exclusion criteria’ were defined as: first, aortic aneurisms as defined by an aorta diameter exceeding 3 cm; second, extensive atherosclerosis as defined by confluent atheromatous plaques or calcifications along the entire aortic circumference; third, visible heart operations in the open heart, such as valve replacement including transcatheter aortic valve operation (TAVR) and coronary artery bypass; fourth, peripheral arterial occlusive disease (Fontaine III–IV), having cause amputation or gangrene; fifth, BMI exciding 30 kg/m^2^.
- ‘Obvious signs of anticoagulation/heparinization’: we defined bruises on belly, fluidity of blood during dissection and absence of clots as obvious signs of anticoagulation.
- ‘Voluminous Adamkiewicz artery’: we defined a radiculomedullary arteries with diameter >1.0 mm, a characteristic hairpin anastomosis to the anterior spinal artery (ASA) as a voluminous Adamkiewicz artery

### Statistical analysis

IBM SPSS Statistics Version 28.0 (IBM Corp., Armonk, NY, USA) was used for descriptive statistics.

## RESULTS

Within a time period of 5 months, the Division of Anatomy received 30 body donors in a time frame of less than 15 hours post mortem. Twenty-two cadavers had to be excluded due to the presence of aorta aneurysm, extensive atherosclerosis, heart operation, peripheral arterial disease, spine surgery or a BMI larger than 30 kg/m^2^. Eight of them (26.7%) met the inclusion criteria and were examined. Two of them showed signs of heparinization.

**Figure 2:**
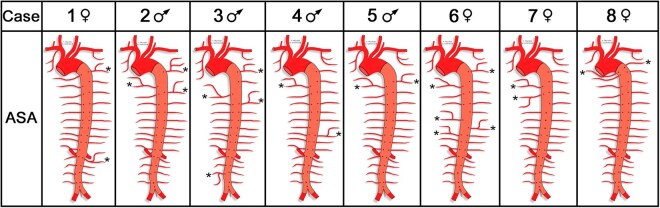
Origin and branching patterns of the aortic segmental arteries. Asterisks (*) indicate variation sites.

All 8 body donors, bilaterally featured 11 left and right posterior intercostal arteries (PIA1–PIA11) and 1 pair of subcostal arteries (SCA). They arose from 15 to 22 TASAs. In 7 body donors (87.5%, 3 females, 4 males), the first TASA emerged at the level of body segment T3 and in 1 (12.5%, 1 female) at the level of T2. Figure 2 informs about other variations.

In all specimens, perfusion via the coeliac artery had stained a continuous section along the cranio-caudal extension of the dura mater and spinal cord. In 6 body donors (75%, 3 females, 3 males), the lumen of the ASA was filled in its full cranio-caudal extension. In 7 specimens (87.5%, 4 females, 3 males), dye had entered the posterior spinal arteries cranial and caudal to the stained segment of the spinal cord and stained the dorsal root fibers.

**Figure 3:**
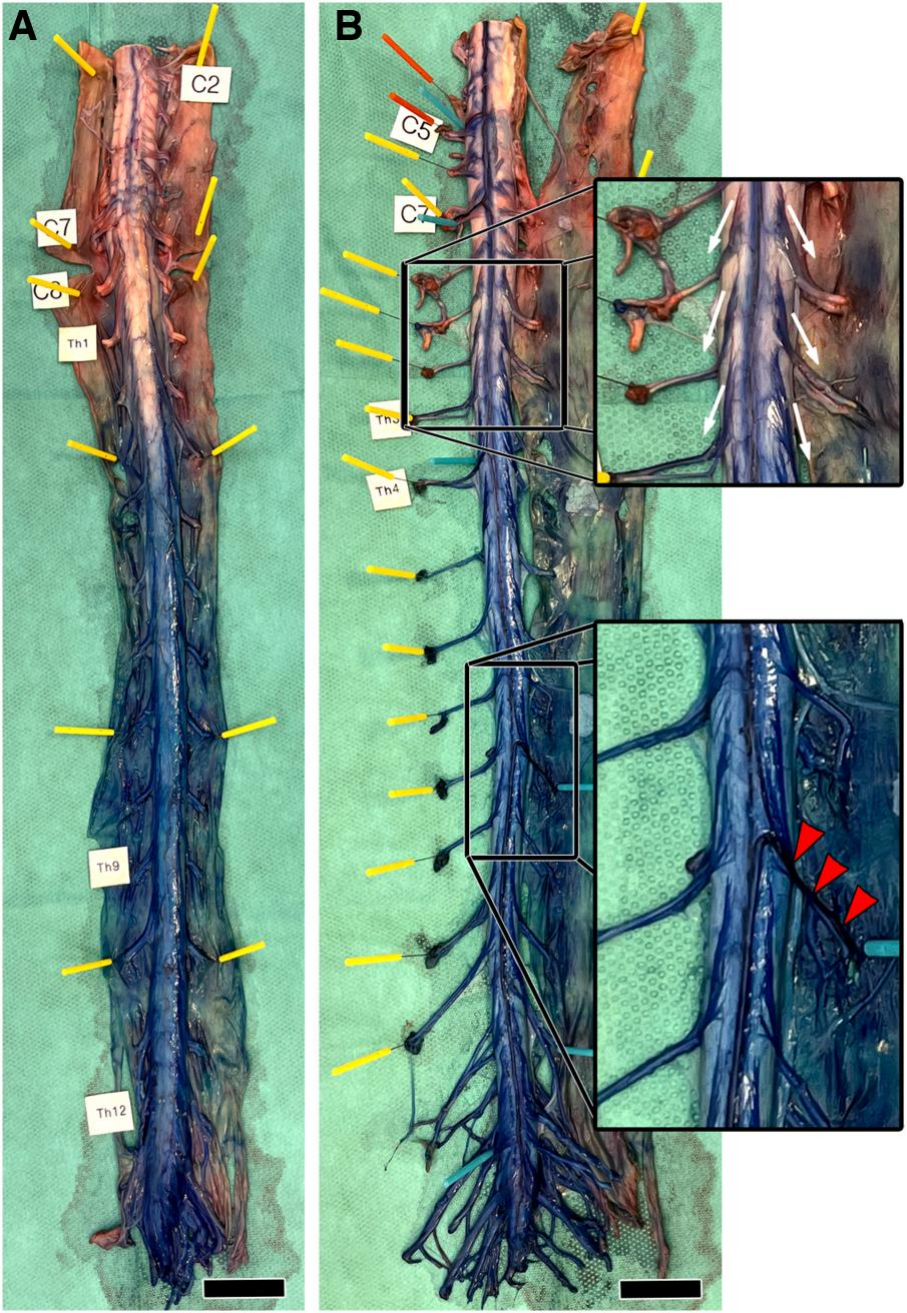
Anterior spinal artery (ASA) and Adamkiewicz artery (arrowheads) and intraspinal angiosomes from posterior (**A**) and anterior (**B**). Note the dye in the posterior spinal arteries at the cervical segments (**A**). Top inlay shows perfusion of anterior nerve root vessels via the ASA (arrows). Bottom inlay shows joining of Adamkiewicz artery with ASA. Note the bifurcation in a caudally and a cranially directed branch. Scale bars, 2 cm.

**Figure 4:**
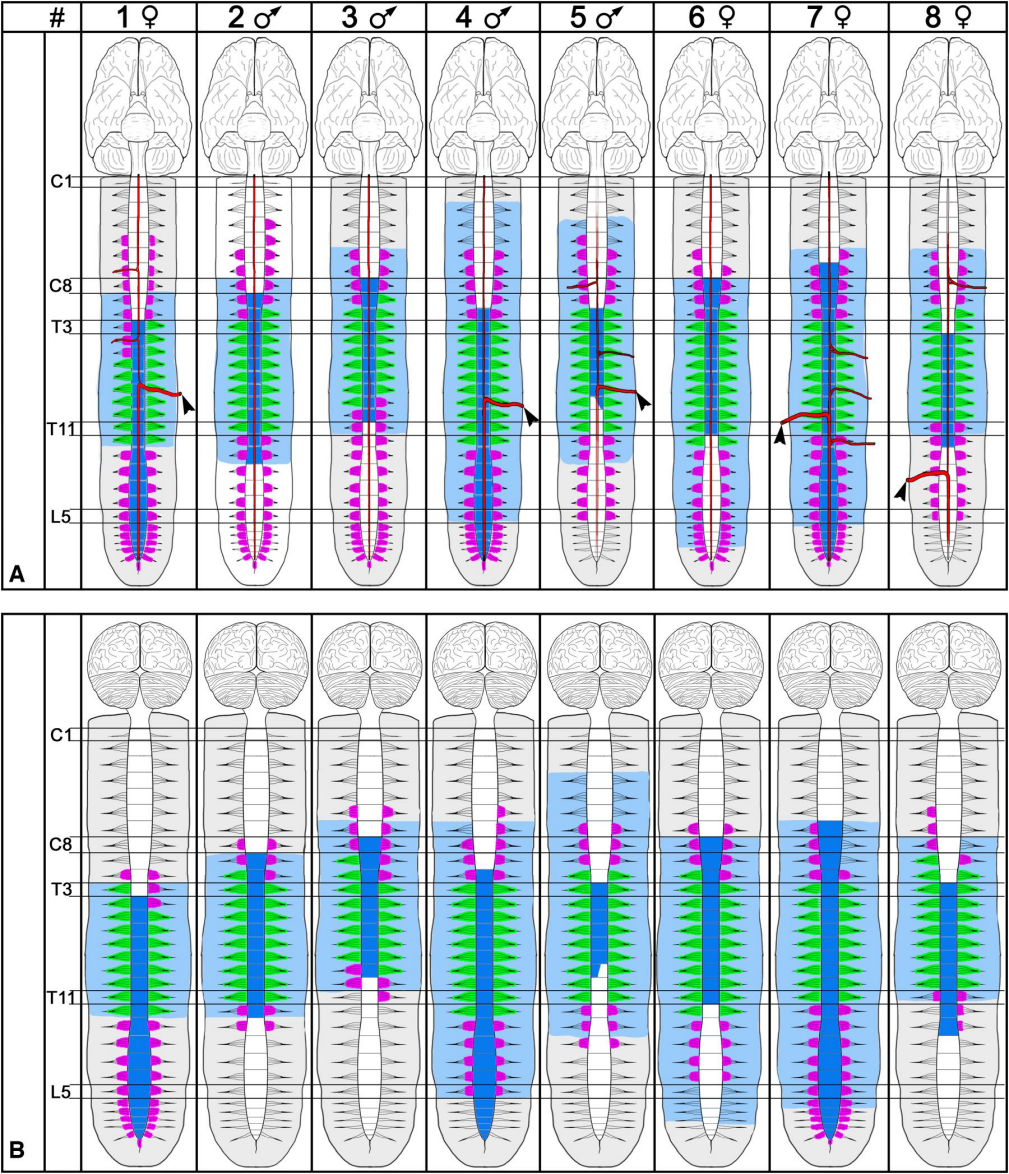
Scheme demonstrating the staining patterns of the examined specimens from anterior (**A**) and posterior (**B**). Light blue, epidural angiosome; deep blue, spinal cord angiosome; green, spinal nerves and associated roots stained via direct spinal artery branches; pink, stained spinal nerve rootlets, stained via branches of the anterior and posterior spinal artery. Red lines in A, stained segment of the anterior spinal artery and Adamkiewicz artery (arrowheads). C: cervical; L: lumbar; T: thoracic.

In 5 specimens (62.5%, 3 females, 2 males), a prominent arteria radicularis magna (Adamkiewicz artery) was identified (Figs 3 and 4). In 4 specimens, it joined the ASA at segments T8 (2 times), T9 and T10. In 1 specimen, it joined the ASA at segment L2 and thus was not perfused by dye injected into the coeliac trunk (Fig. 3).

### Spinal cord angiosome

**Figure 5:**
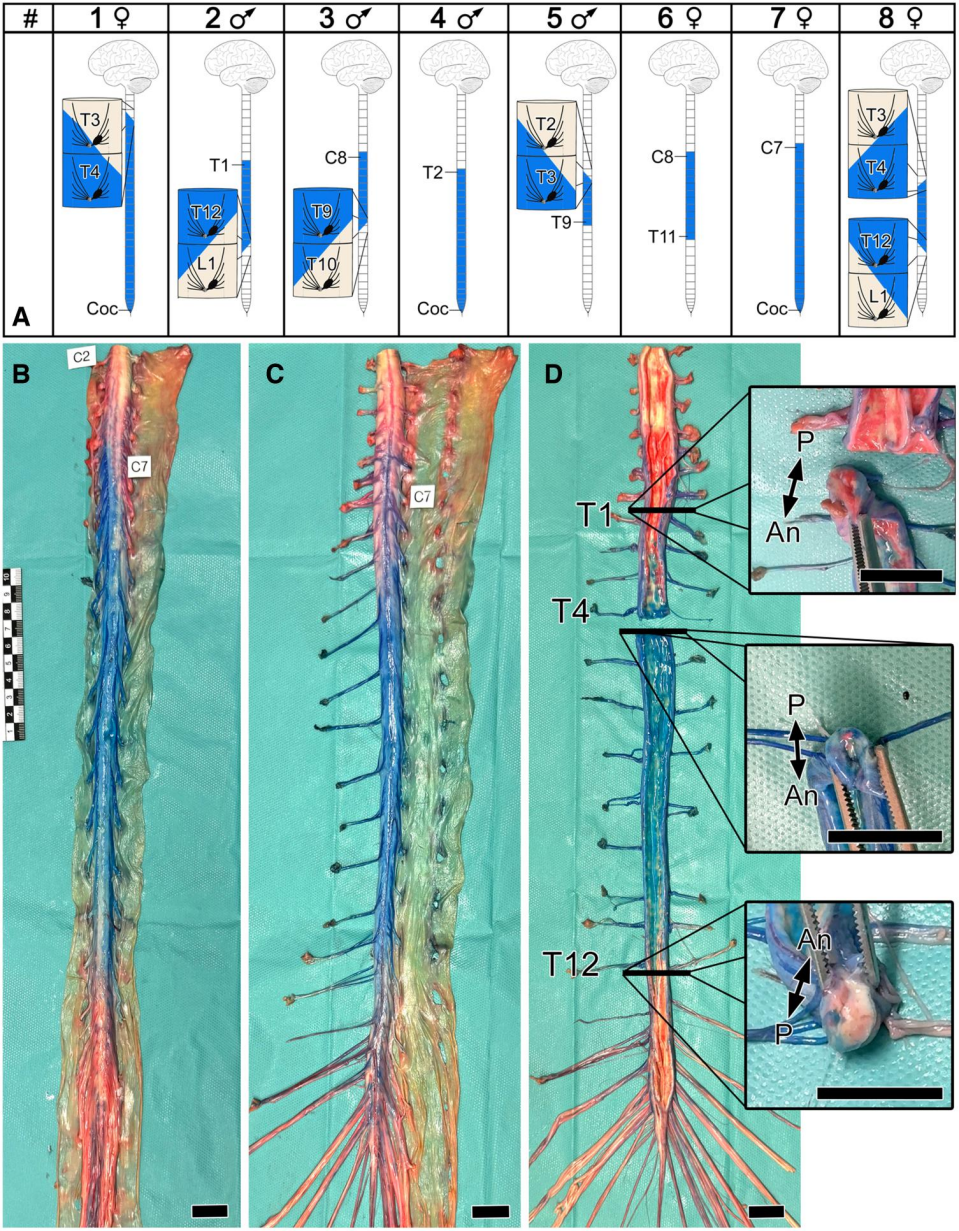
Borders of the spinal cord angiosome. (**A**) Schematic characterization of the cranial and caudal borders. (**B**, **C**). Specimen #8 from posterior (**A**) and anterior (**B**). (**D**) Cranial and caudal borders of specimen #8. Inlays show cross sections of the spinal cord demonstrating the relationship between the anterior and posterior aspect of the spinal cord angiosome at levels thoracic (T)1, T4 and T12. Note the staining pattern of the spinal nerve roots. C: cervical; Coc: coccygeal; L: lumbar. Scale bars, 2 cm.

In 3 body donors (37.5%, 2 females, 1 male), the anterior and posterior aspects of the cranial border, and in 3 (37.5%, 1 female, 2 males) body donors, the anterior and posterior aspect of the caudal border of the stained section of the spinal cord terminated at different segment levels (Figs 4 and 5).

In 2 body donors (25%, 1 female, 1 male), the anterior cranial border was 1 segment more cranially and in 1 body donor (12.5%, 1 female) more caudally than the posterior cranial border. In 2 body donors (25%, 2 males), the anterior caudal border was 1 segment more caudally and in 1 body donor (12.5%, 1 female) 1 segment more cranially than the posterior caudal border.

On average, the cranial border of the anterior and posterior aspect of the spinal cord angiosome was at the level of spinal cord segment T1 (C7–T4); the caudal border at spinal cord segment T12 (T9–Coc.). In 3 cases (37.5%, 2 females, 1 male), the anterior and posterior aspect of the caudal border of the spinal cord angiosome extended caudally until the tip of the conus medullaris. All these specimens featured a voluminous Adamkiewicz artery.

### Nerve root perfusion

The dorsal and ventral spinal nerve root fibers emerging from the spinal cord were stained along the entire spinal cord angiosomes. Additionally, the ventral root fibers of spinal nerves located on average 4 (1–6) segments and the dorsal root fibers of spinal nerves located on average 2 (0–5) segments more cranially than the border of the spinal cord angiosome were stained as well. In the 3 specimens featuring an Adamkiewicz artery and having the spinal cord angiosome extending towards the conus medullaris, the ventral root fibers of all caudal spinal nerves and in 2 specimens also of all dorsal root fibers of the caudal spinal nerves were stained. In the remaining specimen, only the dorsal root fibers of the lumbar spinal nerves were stained, with the sacral ones remaining unstained. In the rest of the specimens, the ventral root fibers of the spinal nerves located on average 6 (0–13) segments and the dorsal root fibers of spinal nerves located on average 2 (0–5) segments more caudally than the caudal border of the spinal cord angiosome were stained. In 7 cases (87.5%, 3 females, 4 males), there was an asymmetrical staining pattern between the left and right-sided ventral and dorsal nerve root fibers (Figs 3–5).

### Epidural (dura mater) angiosome

In 6 body donors (75%, 3 females, 3 males), the cranial borders of the epidural angiosome differed between the ventral and dorsal side (Figs 4 and 5).

On average, the cranial border of anterior aspect of the epidural angiosome was at the level of body segment C6 (C3–T2). Its caudal border was on average at T12 (T11–Sac.) body segment. In 2 specimens (25%, 2 female), it extended caudally into sacral body segments.

On average, the cranial border of the posterior aspect of the dura mater angiosome was at the level of body segment C8 (C4 - T3). Its caudal border was on average at L1 (T10–Sac.) body segment. In 2 specimens (25%, 2 female), it extended caudally into sacral segments (Fig. 4).

## DISSCUSION

Spinal cord ischaemia is a serious complication of open and endovascular surgery of the thoracic aorta [8–14]. In order to explore the causality and to estimate the risk, a remarkable number of studies provide information about the extradural and spinal cord vascularization [3, 15–19]. However, demonstration of the extension of the intraspinal angiosomes of the thoracic aorta is still missing. Our study provides information that may help in clinical practice. Using a cadaver setting, it defines the borders of the angiosomes of the thoracic aorta in regard to the spinal cord and the dura mater under a simulated median pressure of 90 mmHg. It also demonstrates the capacity of intraspinal collaterals, the ‘arteria radicularis magna (ARM)’ or ‘Adamkiewicz artery’ and the ASA and suggests an influence of anatomic variations of the aortic segmental arteries on intraspinal blood supply. In our study, the Adamkiewicz artery was identified in 62.5% of body donor cadavers and demonstrated considerable variability in origin, patterns of anastomosis to the ASA and laterality, which are consistent with previously published literature [15, 20, 21].

In simulation projects, high-quality results can only be obtained by using non embalmed body donors, dissected within a reasonable time frame after death. We therefore decided to restrict our study to only use body donors, immediately after their arrival at our institution in secured 15 hours post mortem. This reduced the number of body donors we could potentially include to 30. In addition, our aim was to simulate spinal cord perfusion in largely normal aged aortae. Since spinal circulation may be altered in impaired, compressed or diseased aortic segmental arteries, we were forced to exclude another 22 with obvious pathologies of the heart, thorax or vascular system.

We decided to simulate the intraoperative conditions of thoracic endovascular aortic repair (TEVAR) procedures preserving the coeliac trunk [22]. Since the coeliac trunk arises at the level of Th12, this includes that the DTA was only perfused distally towards the origin of the 11th posterior intercostal artery. To simulate *in vivo* conditions as closely as possible, we flushed the arteries and then perfused them with a solution that mimics the physical properties of blood and applying a mean pressure of 90 mmHg. Naturally this neglects the influence of individual blood pressure and does not resemble the conditions under maximum systolic pressure. We had to accept this, since we were unable to gain access to information on the *in vivo* blood pressure of each individual we included.

It is highly likely that the tissue volumes perfused in the post-mortem setting with a pressure of 90 mmHg are smaller than *in vivo*. Also, we cannot rule out the presence of microthrombi in small vessels and capillaries, which result in artificial none perfusion of blocked tissue volumes. A specimen, which might have suffered such artificial blockades, is specimen 5. The caudal border of its spinal cord angiosome was at T9, although the specimen had regular aortic segmental arteries down to T11 and the ASA was strengthened by a voluminous Adamkiewicz artery. We therefore like to emphasized that, although we created simulation conditions that very closely resemble the *in vivo* situation, there are inherent artifacts that have to be considered while interpreting the results.

In most specimens with aortic segmental artery variations, the variations seem to have had an effect on the borders of the angiosomes. This strongly underscores the importance of preoperative imaging of the aortic segmental arteries configuration in the planning phase of endovascular and open aorta surgery [2].

Beside the spinal cord angiosomes, we analysed the extension of the extradural (dura mater) angiosome. One motivation was to evaluate the potential of the extradural collateral arterial network [3]. In each individual, the cranial borders of the dura mater angiosome exceeded or at least levelled with the cranial border of the spinal cord angiosome. We consider this as a proof that the epidural collateral network, even if applying only median blood pressure is sufficient to supply blood to the epidural space of adjacent body segments.

The caudal border of the epidural angiosomes usually exceeded or was near the level of the caudal border of the spinal cord angiosomes. A dramatic exception, however, was 3 specimens, in which the spinal cord angiosomes continued towards the medullary conus, while the dura mater angiosomes terminated in the lumbar region. Interestingly, all of these specimens featured an Adamkiewicz artery [23] that joined the ASA in the caudal thorax. This impressively supports the postulate of a dominant role of the ASA and the Adamkiewicz arteries for the perfusion of both, the anterior and posterior aspects of the caudal spinal cord [20, 21, 24]. Preoperative assessment of the capacity of these 2 vessels would definitively allow to estimate the risk of suffering spinal cord ischaemia during aorta surgery.

In all specimens, the lengths of the segment of the ASA that was filled with dyed liquid were substantially longer than the length of the spinal cord angiosomes. In 75%, the ASA was even filled throughout its entire cranio-caudal extension. In addition, in all specimens, the ventral and dorsal root fibers of a significant number of spinal nerves were stained, although these fibers exited from unstained spinal cord segments. In our opinion, this indicates that the pressure applied during perfusion was sufficient to cause liquid to leave the ASA and enter the pial plexus of the majority of the spinal cord segments. The pressure seemed to have been sufficient to also secure flow of liquid in the branches supporting the roots of the spinal nerves in the subarachnoid space. In contrast, the applied pressure seems to have been too low to allow perfusion of branches perforating the dense spinal cord tissue. Consequently, higher pressure would have caused perfusion and staining of the spinal cord tissue. This support concepts, which assign the ASA and sufficient blood pressure an essential role in functional blood supply to the spinal cord and preventing ischaemia [25–28]. Interestingly, in 1 specimen, even the posterior spinal arteries of the entire cervical region and dorsal spinal nerve roots were perfused via collaterals from the ASA. This supports the assumption that blood pressure indeed differs in the branches of the pial plexus, which supply the spinal nerve roots and the spinal cord tissue.

The DTA gives rise to TASAs, which in turn form spinal arteries that enter the intervertebral foramina. Small branches of these arteries supply the spinal nerve, extradural tissue and dura mater and feed into the extradural network of arterial collaterals [3]. We evaluated the staining pattern of the spinal nerves and their associated roots. As expected, at least the entire intraspinal course of spinal nerves and associated roots of T3 to T11, the segments at which TASAs arise from the DTA, was stained. In most specimens, the spinal nerves proofed to be even stained in more cranial and more caudal segments, demonstrating the sufficiency of the epidural collateral network.

Through each intervertebral foramen, spinal arteries enter the spinal canal. Some of them form branches, which proceed into the subdural and subarachnoid spaces and as so called radicular arteries. These ascend with the spinal nerve root fibers towards the level of the corresponding spinal cord segment and supply blood to the ASA [16, 18, 29, 30]. Since, in many segments, the spinal nerves and adjacent segments of their roots are stained, but the corresponding spinal cord segments remain unstained, our results nicely demonstrate that not every spinal artery forms the radiculomedullary artery. Obviously collateral circulation via the ASA at the applied pressure of 90 mmHg is not sufficient to compensate for missing artery. Further perfusion studies comparing the effect of systematically applying various pressure are required to define the exact role of blood pressure on spinal cord collateral perfusion. Yet, even our results indicate that increasing blood pressure during aorta surgery, at least for spinal cord perfusion, might be beneficial.

### Limitations

To sum up the most important limitations: first, for technical reasons, we were forced to accept to examine only a total of 8 cadavers. This small sample size limits the representativeness of the findings and their generalizability to the wider patient population; second, the individual patterns of the segmental arteries do not represent the full spectrum of possible variations; third, our post-mortem perfusion was undertaken without acknowledging physiologic blood flow, pulsatility, vascular tone and normal blood rheology in body donor specimens may influence the distribution of perfusion areas.

## CONCLUSION

In a nutshell, we provide precise anatomic information on the extension of the 2 intraspinal angiosomes at a simulated mean arterial pressure of 90 mmHg. However, further studies, applying different pressure levels, are required to evaluate the true potential of blood pressure increase for preventing spinal ischaemia during aorta surgery.

## Data Availability

Data are available upon reasonable request.

## ABBREVIATIONS

ASA: Anterior spinal artery
BMI: Body mass index
DTA: Descending thoracic aorta
TASA: Thoracic aortic segmental artery

## References

[1] Czerny M , Grabenwöger M, Berger T et al; EACTS/STS Scientific Document Group. EACTS/STS guidelines for diagnosing and treating acute and chronic syndromes of the aortic organ. Eur J Cardiothorac Surg 2024;118:5–115. 10.1016/j.athoracsur.2024.01.021 38416090

[2] Pruidze P , Weninger JT, Didava G et al Anatomy of the aortic segmental arteries-the fundamentals of preventing spinal cord ischemia in aortic aneurysm repair. Front Cardiovasc Med 2024;11:1475084. 39691497 10.3389/fcvm.2024.1475084 PMC11649645

[3] Heber UM , Mayrhofer M, Gottardi R et al The intraspinal arterial collateral network: a new anatomical basis for understanding and preventing paraplegia during aortic repair. Eur J Cardiothorac Surg 2021;59:137–44. 32710104 10.1093/ejcts/ezaa227

[4] Taylor GI , Palmer JH. The vascular territories (angiosomes) of the body: experimental study and clinical applications. Br J Plast Surg 1987;40:113–41. 3567445 10.1016/0007-1226(87)90185-8

[5] Hirtler L , Reissig L, Weninger WJ. Sezieranleitung für Organmorphologie II. © 2024 Abteilung für Anatomie, Wien: Medizinische Universität Wien, 2024.

[6] Reissig L , Hirtler L, Weninger WJ. Sezieranleitung für Organmorphologie I. © 2024 Abteilung für Anatomie, Wien: Medizinische Universität Wien, 2024.

[7] Polanczyk A , Klinger M, Nanobachvili J, Huk I, Neumayer C. Artificial circulatory model for analysis of human and artificial vessels. App Sci 2018;8:1017.

[8] Amabile P , Grisoli D, Giorgi R, Bartoli JM, Piquet P. Incidence and determinants of spinal cord ischaemia in stent-graft repair of the thoracic aorta. Eur J Vasc Endovasc Surg 2008;35:455–61. 18180183 10.1016/j.ejvs.2007.11.005

[9] Awad H , Ramadan ME, El Sayed HF, Tolpin DA, Tili E, Collard CD. Spinal cord injury after thoracic endovascular aortic aneurysm repair. Can J Anaesth 2017;64:1218–35. 29019146 10.1007/s12630-017-0974-1 PMC5954412

[10] Buth J , Harris PL, Hobo R et al Neurologic complications associated with endovascular repair of thoracic aortic pathology: incidence and risk factors. a study from the European Collaborators on Stent/Graft Techniques for Aortic Aneurysm Repair (EUROSTAR) registry. J Vasc Surg 2007;46:1103–10; discussion 10-1. 18154984 10.1016/j.jvs.2007.08.020

[11] Gaudino M , Khan FM, Rahouma M et al Spinal cord injury after open and endovascular repair of descending thoracic and thoracoabdominal aortic aneurysms: a meta-analysis. J Thorac Cardiovasc Surg 2022;163:552–64. 32561196 10.1016/j.jtcvs.2020.04.126

[12] Khoynezhad A , Donayre CE, Bui H, Kopchok GE, Walot I, White RA. Risk factors of neurologic deficit after thoracic aortic endografting. Ann Thorac Surg 2007;83:S882–9; discussion S90-2. 17257946 10.1016/j.athoracsur.2006.10.090

[13] Sotir A , Klopf J, Brostjan C, Neumayer C, Eilenberg W. Biomarkers of spinal cord injury in patients undergoing complex endovascular aortic repair procedures-a narrative review of current literature. Biomedicines 2023;11(5):1317. 10.3390/biomedicines11051317 PMC10216375 37238988

[14] Upchurch GR , Escobar GA, Azizzadeh A et al Society for Vascular Surgery clinical practice guidelines of thoracic endovascular aortic repair for descending thoracic aortic aneurysms. J Vasc Surg 2021;73:55s–83s. 32628988 10.1016/j.jvs.2020.05.076

[15] Alvernia JE , Simon E, Khandelwal K et al Anatomical study of the thoracolumbar radiculomedullary arteries, including the Adamkiewicz artery and supporting radiculomedullary arteries. J Neurosurg Spine 2023;38:233–41. 36152330 10.3171/2022.5.SPINE2214

[16] Lazorthes G , Gouaze A, Zadeh JO, Santini JJ, Lazorthes Y, Burdin P. Arterial vascularization of the spinal cord: recent studies of the anastomotic substitution pathways. J Neurosurg 1971;35:253–62. 22046635 10.3171/jns.1971.35.3.0253

[17] Morishita K , Murakami G, Fujisawa Y et al Anatomical study of blood supply to the spinal cord. Ann Thorac Surg 2003;76:1967–71. 14667623 10.1016/s0003-4975(03)01254-2

[18] Santillan A , Nacarino V, Greenberg E, Riina HA, Gobin YP, Patsalides A. Vascular anatomy of the spinal cord. J Neurointerv Surg 2012;4:67–74. 21990489 10.1136/neurintsurg-2011-010018

[19] Vuong SM , Jeong WJ, Morales H, Abruzzo TA. Vascular diseases of the spinal cord: infarction, hemorrhage, and venous congestive myelopathy. Semin Ultrasound CT MR 2016;37:466–81. 27616317 10.1053/j.sult.2016.05.008

[20] Koshino T , Murakami G, Morishita K, Mawatari T, Abe T. Does the Adamkiewicz artery originate from the larger segmental arteries? J Thorac Cardiovasc Surg 1999;117:898–905. 10220681 10.1016/S0022-5223(99)70369-7

[21] Taterra D , Skinningsrud B, Pękala PA et al Artery of Adamkiewicz: a meta-analysis of anatomical characteristics. Neuroradiology 2019;61:869–80. 31030251 10.1007/s00234-019-02207-y PMC6620248

[22] Falkenberg M , Lönn L, Schroeder T, Delle M. TEVAR and covering the celiac artery. Is it safe or not? J Cardiovasc Surg (Torino) 2010;51:177–82. 20354487

[23] Adamkiewicz A. Die Blutgefässe des menschlichen Rückenmarkes. I. Theil. Die Gefässe der Rückenmarksubstanz. II. Theil. Die Gefässe der Rückenmarksoberfläche. Sitzungsberichte Der Akad Der Wissenschaften Wien (Math-Naturwiss Classe) 1881;84:469–502.

[24] Melissano G , Bertoglio L, Rinaldi E, Leopardi M, Chiesa R. An anatomical review of spinal cord blood supply. J Cardiovasc Surg (Torino) 2015;56:699–706. 25881616

[25] Dijkstra ML , Vainas T, Zeebregts CJ, Hooft L, van der Laan MJ. Editor’s choice—spinal cord ischaemia in endovascular thoracic and thoraco-abdominal aortic repair: review of preventive strategies. Eur J Vasc Endovasc Surg 2018;55:829–41. 29525741 10.1016/j.ejvs.2018.02.002

[26] Grabenwöger M , Alfonso F, Bachet J et al Thoracic endovascular aortic repair (TEVAR) for the treatment of aortic diseases: a position statement from the European Association for Cardio-Thoracic Surgery (EACTS) and the European Society of Cardiology (ESC), in collaboration with the European Association of Percutaneous Cardiovascular Interventions (EAPCI). Eur J Cardiothorac Surg 2012;42:17–24. 22561652 10.1093/ejcts/ezs107

[27] Kwon BK , Tetreault LA, Martin AR et al A clinical practice guideline for the management of patients with acute spinal cord injury: recommendations on hemodynamic management. Global Spine J 2024;14:187s–211s. 38526923 10.1177/21925682231202348 PMC10964888

[28] Lella SK , Waller HD, Pendleton A, Latz CA, Boitano LT, Dua A. A systematic review of spinal cord ischemia prevention and management after open and endovascular aortic repair. J Vasc Surg 2022;75:1091–106. 34740806 10.1016/j.jvs.2021.10.039

[29] Gillilan LA. The arterial blood supply of the human spinal cord. J Comp Neurol 1958;110:75–103. 13631126 10.1002/cne.901100104

[30] Turnbull IM. Microvasculature of the human spinal cord. J Neurosurg 1971;35:141–7. 5570776 10.3171/jns.1971.35.2.0141

